# Gastrointestinal decontamination in the acutely poisoned patient

**DOI:** 10.1186/1865-1380-4-65

**Published:** 2011-10-12

**Authors:** Timothy E Albertson, Kelly P Owen, Mark E Sutter, Andrew L Chan

**Affiliations:** 1 Department of Internal Medicine, School of Medicine, University of California, Davis, Sacramento, California, USA; 2 Department of Emergency Medicine, School of Medicine, University of California, Davis, Sacramento, California, USA

## Abstract

**Objective:**

To define the role of gastrointestinal (GI) decontamination of the poisoned patient.

**Data Sources:**

A computer-based PubMed/MEDLINE search of the literature on GI decontamination in the poisoned patient with cross referencing of sources.

**Study Selection and Data Extraction:**

Clinical, animal and in vitro studies were reviewed for clinical relevance to GI decontamination of the poisoned patient.

**Data Synthesis:**

The literature suggests that previously, widely used, aggressive approaches including the use of ipecac syrup, gastric lavage, and cathartics are now rarely recommended. Whole bowel irrigation is still often recommended for slow-release drugs, metals, and patients who "pack" or "stuff" foreign bodies filled with drugs of abuse, but with little quality data to support it. Activated charcoal (AC), single or multiple doses, was also a previous mainstay of GI decontamination, but the utility of AC is now recognized to be limited and more time dependent than previously practiced. These recommendations have resulted in several treatment guidelines that are mostly based on retrospective analysis, animal studies or small case series, and rarely based on randomized clinical trials.

**Conclusions:**

The current literature supports limited use of GI decontamination of the poisoned patient.

## Introduction

In the United States, the American Association of Poison Control Centers (AAPCC) reported about 2.4 million poisoning exposures a year in 2006, while the Institute of Medicine in 2001 estimated more than 4 million poisoning episodes with 300,000 hospitalizations and 24,173 poisoning-related deaths [1-3]. This article will review the current recommendations, guidelines and data on gastrointestinal (GI) decontamination in the poisoned patient.

Gastrointestinal decontamination of the poisoned patient has evolved significantly over the last 2 1/2 decades from a very invasive to a less aggressive approach. This less aggressive approach to GI decontamination followed a series of position statements published jointly by the American Academy of Clinical Toxicology (AACT) and the European Association of Poison Centres and Clinical Toxicologists (EAPCCT) in the late 1990s into the early 2000s [4-9]. Despite the publication of these guidelines and several outstanding reviews [10-12], data suggest that few clinicians have read the guidelines and many have poor knowledge about the use of GI decontamination [13]. In addition, uniform advice on this topic has not been demonstrated from poison information centers [14].

### Criteria for selection and assessment of literature

An extensive PubMed/MEDLINE search for gastrointestinal decontamination, activated charcoal (AC), multiple dose activated charcoal (MDAC), ipecac, ipecac syrup, gastric emptying (GE), gastric lavage (GL), whole bowel irrigation (WBI), body packers, body stuffers and poisoning treatments was done from about 1980 to present. Specific drugs including acetaminophen, anticonvulsants, N-acetylcysteine, theophylline, salicylic acid/aspirin, digoxin, yellow oleander and isoniazid were searched for relevant studies related to AC and poisoning. Human clinical trials with randomized GI decontamination treatments and outcome data were given the highest priority in the review.

### Gastrointestinal decontamination/ipecac

In the late 1970s and early 1980s, the use of ipecac syrup to induce vomiting after oral poisoning was widespread both at home and in the hospital [15]. Earlier work had suggested it was safer than the parenterally dosed apomorphine [16]. Ipecac contains alkaloids from the plants *cephalis acuminata *and *cephalis ipecacuanha*. The active compounds of these plants include emetine and cepheline, which cause emesis by local gastric irritation and stimulation of the chemotrigger zone of the brain. The most common side effects from ipecac include multiple episodes of vomiting persisting longer than 60 min, aspiration pneumonia, bronchospasm, Mallory-Weiss tears of the esophagus, bradycardia and barotrauma to the mediastinum.

In a simulated overdose study of acetaminophen, ten healthy subjects ingested 3 g of acetaminophen followed by no intervention, ipecac or 50 g AC-sorbitol solution at 1 h with acetaminophen levels being repeatedly drawn [17]. Both treatments significantly (*P *≤ 0.05) reduced serum acetaminophen levels compared to control, but no differences between ipecac or AC treatments were seen. Using a simulated salicylate model, 12 adult volunteers took 24 81-mg aspirin tablets and were randomly treated as controls or with ipecac, AC plus magnesium sulfate or ipecac plus AC plus magnesium sulfate [18]. Total urinary salicylate excretion found 96.3% ± 7.5% of the salicylate in the control, 70.3% ± 11.8% after ipecac and 56.4%± 12% after AC plus magnesium sulfate. Only eight of ten patients who received ipecac plus AC plus magnesium sulfate were able to retain the AC, making analysis of this group impossible. The use of AC alone significantly reduced the recovered salicylate compared to both control (*P *< 0.01) and ipecac groups (P *<*0.01), while ipecac treatment significantly reduced (*P *< 0.01) recovered salicylate levels compared to control [18].

In a series of four randomized trials, GE with ipecac for alert patients or GL for obtunded patients was followed by AC and compared to AC alone [19-22]. No improvement in outcomes was seen when GE approaches plus AC treated patients were compared to AC treatment alone. Three out of four of the studies reported higher complication rates with GE procedures, with aspiration pneumonia being the most predominant [19-21]. Aspiration pneumonia was more common after combined GE and AC treatment than with AC alone [20,21]. An unblinded trial of children less than 6 years old who presented with mild to moderate acute oral ingestions were randomized to ipecac followed by AC versus AC alone [23]. There was no significant difference in clinical outcomes reported in the 70 patients randomized, but it took significantly longer to receive the AC when ipecac was given (2.6± 0.1 h vs. 0.9± 0.1 h, *P *< 0.05). As a result, those receiving ipecac spent significantly longer time in the emergency department (ED) (4.1± 0.2 h vs. 3.4± 0.2 h, *P *< 0.05). Home use of ipecac also has failed to reduce ED use or to improve outcomes in a cohort comparison [24].

The combined position statement of the AACT and EAPCCT concluded that there was no evidence from clinical studies that ipecac improves the outcome of poisoned patients and its routine administration in the ED should be abandoned [4]. This was followed by a guideline released by the AAPCC that suggested ipecac should only be used at home if there would be a delay of 1 h or more before a patient could get to an ED and then only if it could be administered within 30-90 min after the ingestion [25]. The American Academy of Pediatrics recommended removing all ipecac from the home setting and that ipecac should not be used routinely as a home treatment for pediatric poisonings [26]. A dramatic reduction in the use of ipecac in the poisoned patient has resulted. In 1985, 14.99% (132,947) of human poison exposures reported to the AAPCC received ipecac, but by 2009 only 0.02% (658) received ipecac [27].

### Gastrointestinal decontamination/gastric lavage

The use of GL for patients with oral poisons who were obtunded or with altered mental status was a standard approach until the 1990s. Matthew et al. [28] in 1966 noted that data from as early as 1942 had questioned the efficacy of GL, but after studying 259 patients who had ingested a poison, he concluded that when GL was performed within 4 h after ingestion, significant recovery of barbiturates in the lavage fluid could still be demonstrated.

As noted before, various GE procedures were studied in acute poisoning cases in the five previously mentioned studies that also included ipecac treatment arms [19-23]. Specifically, GL used for obtunded patients in three of the trials [19,21,22] was compared to AC treatment alone. Two of the studies found no significant improvement in clinical outcome with GL compared to AC [21,22]. One study [19] noted that obtunded, poisoned patients who had GL plus AC within 1 h of ingestion had significantly (*P *< 0.05) less deterioration compared to AC treatment alone. The use of GL plus AC was associated with a significantly (*P *= 0.0001) increased incidence of aspiration pneumonia in one study compared to AC alone in symptomatic patients (8.5% vs. 0.0%) [21]. In a review of four studies that evaluated GL versus AC in acetaminophen overdoses, no strong evidence was found for the routine use of GL [29].

A study of volunteers who had a 34-French orogastric tube placed after acetaminophen ingestion reported that GL within 1 h resulted in a significant reduction in acetaminophen bioavailability [30]. Another study of patients who presented within 4 h of acetaminophen ingestion were randomized to no intervention, GL, AC or treatment with ipecac [31]. A significant reduction in serum acetaminophen levels was seen with ipecac, GL or AC treatments compared to no treatment [31]. Use of AC within 4 h of ingestions resulted in the greatest decrease in acetaminophen levels, greater than those seen with both the GL (*P *= 0.013) and the ipecac syrup treatment groups (*P *= 0.027). Despite those acetaminophen serum reductions, no clinical outcome differences between the groups were reported [31]. A review of two small trials of volunteers with simulated aspirin/non-steroidal anti-inflammatory drug overdoses found no advantage to GL over AC alone [32].

In a study of patients with tricyclic antidepressant overdoses, treatment was randomized to AC, saline GL followed by AC or AC followed by saline GL followed by AC [33]. All three approaches had similar clinical outcomes with no advantage to GL. A systematic review of controlled trials of GL in acute organophosphorus pesticide poisoning found no "high-quality" evidence to support the use of GL in treating these poisonings [34].

One theoretical concern with the use of GL is the potential of flushing drugs out of the stomach into the small intestines. A systematic review of this topic failed to find published data that supported this concern [35]. The major complications with GL include perforation of the esophagus and stomach, pulmonary aspiration and aspiration pneumonia. In an observational study of 14 consecutive GLs performed in four hospitals in Sri Lanka, Eddleston et al. observed five aspirations and two major cardiac events during the procedure [36]. A recent study from India of 98 pesticide-poisoned patients found aspiration pneumonia in 2.2-13.2% of the patients depending on whether the lavage occurred in the academic or referral hospital [37]. A drop in arterial saturations was seen in 13.3%, and laryngospasm, tachycardia, electrolyte imbalance or tube stuck in throat was each at 2.2% [37]. Although these may be higher complication rates than expected in modern health care facilities, it draws a warning about the potential risk of GL [36].

Significant limitations exist in calculating the power needed to show treatment differences in clinical trials related to GE and the treatment of actual poisoned patients. Patients take different combinations of drugs, different amounts, at different times and have different co-morbidities, which all confound the potential ability of a treatment like GE from altering the clinical course. These limitations must be considered when interpreting the results of treatments like GE in studies using actual poisoned patients.

In a review, Bond [38] notes that GL with AC should be considered for symptomatic patients who present within 1 h, who have ingested agents that slow gastrointestinal motility, who have ingested sustained-release medication or who have ingested massive/life-threatening amounts of a medication. This recommendation is in contrast to the 1997 AACT/EPCCT guidelines, which state that GL should not be used for routine poisonings, as there is no certainty that it improves clinical outcomes. Further, the guidelines suggest GL should only be considered in a potentially life-threatening ingestion and then only if it can be undertaken within 60 min of ingestion [5]. Of the over 2.4 million AAPCC reported poisonings in 2009, only 6,093 (0.25%) of the total patients and only 238 (0.02%) of those patients ≤ 5 years of age underwent GL [27].

### Gastrointestinal decontamination/cathartics

Non-absorbable sugars such as sorbitol and salts including magnesium citrate and sodium sulfate have been used as adjuncts to AC to hasten the elimination of the poison-charcoal complex. One experimental trial reported reduced systemic absorption of aspirin, but not pentobarbital, chlorpheniramine or chloroquine when AC was given with sodium sulfate [39]. Activated charcoal alone or in combination with sodium sulfate were thought to be effective, and both approaches reduced the absorption of drugs compared to sodium sulfate alone [39]. Using healthy volunteers in a simulation of an aspirin overdose, AC was more effective than water alone in reducing salicylate absorption as measured by the area under the concentration curve (AUC) (428.24± 218.64 μg/ml·h vs. 846.54± 292.95 μg/ml·h, *P *< 0.01) [40]. Activated charcoal with sodium sulfate had no added effect in reducing aspirin absorption and did not significantly reduce the AUC compared to water alone (618.61± 324.71 μg/ml·h vs. 846.54± 292.95 μg/ml·h, NS) [40]. The authors concluded that adding sodium sulfate to AC did not improve the efficacy of AC.

A survey of EDs in 1991 found that the combination product of AC plus sorbitol was frequently the only AC product stocked in EDs [41]. Even when it was not the only available preparation, 49% of EDs reported giving repeat doses of AC and sorbitol routinely when the MDAC approach was used [42]. Cathartic use is associated with diarrhea, and when multiple doses of cathartics are given, significant free water loss and dehydration can occur. Three cases of severe hypernatremia were reported with magnesium citrate cathartic use in the management of overdoses [43].

The 1997 AACT/EAPCCT position statement notes that cathartics alone have no role in the management of the poisoned patient [6]. Further, the routine use of a cathartic in combination with AC was not endorsed [6]. The 2009 AAPCC report found that 22,034 (0.89%) of calls regarding poisoned patients were treated with cathartics, and most were used with AC [27].

### Gastrointestinal decontamination/whole bowel irrigation (WBI)

Many substances are not fully bound by AC. These include slow-release medications, electrolytes and metals. In an attempt to reduce GI drug transit time, polyethylene glycol (PEG) electrolyte solution with rates of 0.5 to 2.0 l/h have been given to poisoned patients to reduce transit time of the toxic material, to get the poison beyond the GI areas of drug absorption and eventually to be eliminated. The goal of WBI is to remove the substance from the GI tract prior to it being absorbed.

Both simulated overdose or nontoxic exposure studies and clinical overdose cases have questioned the efficacy of WBI particularly in combination with MDAC [41,44-47]. In vitro data have demonstrated adsorption of PEG by AC, which in turn reduced the adsorption of salicylic acid by AC [48]. These investigators questioned the use of AC and WBI together in treating overdoses [48]. No randomized controlled trials have been reported evaluating the efficacy of WBI.

Using enteric-coated aspirin, ten adult volunteers received AC combined with sorbitol, water alone (control) or WBI. Both WBI and the AC with sorbitol significantly (*P *< 0.001) reduced absorption as determined by the salicylic acid AUC. Whole bowel irrigation demonstrated the greatest reduction in salicylic acid AUC compared to AC (*P *< 0.05) and was associated with fewer adverse effects [49]. In another study, nine healthy patients received sustained-release preparations of carbamazepine, theophylline and verapamil followed 1 h later by AC, AC with WBI or water (control) [50]. Activated charcoal reduced the absorption as measured by the serum AUC by 62%-75% for all three drugs compared to control, but the addition of WBI to the AC actually significantly (*P *< 0.01) reduced the efficacy of the AC with carbamazepine (AC, 62% reduction in AUC vs. WBI/AC, 41% reduction in AUC). Similarly, the AUC for theophylline with WBI/AC resulted in less reduction in AUC compared to AC alone (65% vs. 75% reduction in AUC, *P *< 0.001) [50]. In contrast the combination of WBI/AC resulted in a greater reduction of verapamil absorption (85% reduction in AUC) compared to AC alone (63% reduction in AUC, *P *< 0.001) [50]. A study of anesthetized dogs given sustained-release theophylline and treated with either AC, WBI followed by AC or AC followed by WBI failed to demonstrate further reductions in theophylline absorption when WBI was added to the treatment [51].

The most common complication with WBI is pulmonary aspiration. Contraindications for its use include compromised airway, hemodynamic instability, seizures, and the lack of bowel sounds or a suspected or documented bowel obstruction [52].

Though many of the simulated poison studies have not demonstrated a benefit of WBI with or without AC, several case reports note improved outcomes using WBI for unusual exposures including slow-release medications such as theophylline, mercuric oxide, lead, strontium ferrite, potassium capsules and even lead bullets [53-58]. These case reports obviously lack controls or even historic controls, drawing into question whether improved outcomes were really demonstrated.

The 1997 AACT/EAPCCT position statement recommended that WBI not be used routinely in the management of the poisoned patient. Based only on volunteer studies and without randomized clinical studies, the paper recommends that WBI be considered for potentially toxic ingestions of sustained-release or enteric-coated drugs. Because of the lack of data, WBI remains a "theoretical" option for iron, lead or zinc poisonings, or for packets of illicit drugs [59]. A follow-up position paper in 2004 concluded "no new evidence" existed that would require a revision of the previous statement on WBI [60]. It would appear that WBI has a very limited role in the treatment of the poisoned patient. Only 2,108 (0.09%) of total patients and 130 (0.01%) of those patients ≤ 5 years old received WBI out of the more than 2.4 million total poisoned patients reported in 2009 to the AAPCC [27].

### Gastrointestinal decontamination/special consideration/body packers/body stuffers

Body packers or "mules" are people who smuggle illicit drugs (mostly cocaine or opium/heroin) concealed in the mouth, rectum, vagina, ear, foreskin or gastrointestinal tract. The drugs are often packaged in capsules, condoms, balloons or plastic bags. Drugs in most of the anatomical body cavities or orifices can be manually removed except when they have been ingested. Once in the GI tract, decontamination or removal can be challenging, and perforations of the drug container can be fatal. A variation of the concept of the body packer is the "body stuffer" who ingests drugs in aluminum foil or plastic bags often hastily to avoid police seizure of the drug/evidence. A further variation of "body stuffing" is "parachuting" in which the illicit drug (often methamphetamine or cocaine) is swallowed in a plastic baggie that has been punctured multiple times to allow a slow-release of the drug [61]. Adults are usually involved in cases of body packing/stuffing, but disturbing reports of pediatric body packers have surfaced [62].

A recent review of approaches to GI decontamination in these patients noted no randomized clinical trials exist, leaving only case studies and series available to guide our approach [63]. One European series from Paris and Frankfurt included approximately 4,660 body packers. The vast majority of the body packers were asymptomatic. About 1.4% of the body packers (*n *= 64) had life-threatening symptoms of cocaine overdose after rupture or leakage of a container. Forty-four of these body packers died before surgical intervention to remove the packets could be performed, while 20 underwent emergency laparotomy for the removal of the containers and all of these patients survived [64]. Urgent surgery is routinely recommended for symptomatic patients with obstruction, bowel perforation or evidence of drug packet rupture [63-66].

Although surgery has been considered definitive in these cases, a report noted two cases where, despite careful surgical inspection and palpation of the entire GI tract, additional drug packets were identified after surgery by CT scans [67]. Plain abdominal radiographs are reported to have sensitivity for finding drug packets of between 85-90% [68-70]. Contrast-enhanced abdominal radiography has been reported to increase sensitivity to 96% [68,71]. Although no large studies have been done with abdominal CT scanning, its use has been widely reported with CT window manipulation, identifying packets that would not have been seen on routine windows [68,72]. Unfortunately, at least two cases of drug packets missed by CT scanning have been reported [67,73].

With little data, conservative non-surgical measures are routinely used in the majority of patients who are asymptomatic. These approaches include endoscopy for packets thought to be retained in the stomach, light solid diets, oral fluids, AC and most commonly WBI to clear the GI tract of the drug packets [68]. Oil-based laxatives have been recommended in the past, but are now discouraged because of their potential effect on latex products used to package some drugs [68,74]. The packets can often be collected after sorting through stool in a way that preserves them as evidence [61,75-77]. Although not formally tested, the value of a systematic surgical protocol for treating body packers that defines conservative therapy, monitoring approaches, as well as when to use surgery has been emphasized [78,79].

### Sodium polystyrene sulfonate

The use of sodium polystyrene sulfonate (SPS) has been recommended in the treatment of lithium overdoses to prevent further absorption and potentially increase lithium elimination [80]. The use of SPS for the treatment of hyperkalemia has been clinically widespread since its approval by the FDA in 1958. Recently, the efficacy and safety of SPS for hyperkalemia have been questioned [81].

In vitro binding of lithium to SPS has been demonstrated [82]. Mice studies have shown significant reduction in lithium levels after oral lithium is followed by SPS. These reductions have been shown after multiple doses of SPS and with delays up to 90 min after the oral dosing of lithium, while treatment with AC has shown no effect [83-86]. Human subjects exposed to 900 mg lithium were treated 30 min later with 0, 20 or 40 g of SPS. Reduced absorption and increased clearance of lithium were demonstrated with SPS [87]. Two additional human volunteer studies have confirmed reduced lithium serum AUC, lower peak levels and delayed time to peak lithium levels when SPS is given after oral lithium [88,89]. A retrospective cohort study of patients with lithium toxicity treated with oral SPS suggested more than a 50% reduction in lithium serum half-life. No clinical outcome improvement was reported. Constipation and mild hypokalemia were noted as side effects of treatment in this study [90].

Hypokalemia is an expected complication of the use of SPS for lithium overdose, but more troubling are reports suggesting GI necrosis in uremic patients treated with SPS [91,92]. The use of sorbitol to prevent fecal impaction and to increase GI motility as an osmotic cathartic with the SPS powder or as a pre-mixed product has been suggested to be related to the development of GI necrosis [81]. Cases of calcium polystyrene sulfonate without sorbitol-associated GI necrosis exist and bring into question the importance of sorbitol to the mechanism [93,94]. The incidence of this severe side effect is not currently known, and to date no cases of GI necrosis have been reported as a result of using SPS for the treatment of lithium-overdosed patients.

### Gastrointestinal decontamination/activated charcoal

A dramatic early demonstration of AC occurred when Tovery, before the 1831 French Academy, took a lethal dose of strychnine mixed with charcoal. He suffered no ill effects [10,95,96]. Activated charcoal is a highly adsorbent material produced by grinding carbon material into a fine powder, superheating the carbon source to very high temperatures, followed by injecting or "activating" the charcoal with steam to maximize the surface area. By the 1980s, AC was generally recognized as a major treatment for most orally poisoned patients [95].

In addition to preventing drug absorption, AC can disrupt drug recycling in an enterohepatic loop and results in "drug back diffusion" out of the body into the GI tract. This "gut dialysis," where drugs move from systemic body compartments to the GI tract, explains how drugs given intravenously can have increased elimination with oral AC. Many trials have documented increased clearance with oral AC in both human and animal models after intravenous (IV) phenobarbital, phenytoin, digoxin, acetaminophen and theophylline (Table 1). These proposed AC mechanisms go beyond traditional GI decontamination and actually invoke a process that increases elimination. Multiple human and animal studies have suggested that MDAC is potentially an important treatment approach for many toxins including some IV poisonings, slow release compounds, pill bezoars or massive poisonings. In the early 2000s, some review articles were still supportive of the use of AC and MDAC (usually without GE), but others began to question this practice [38,97]. Table 1 offers in-depth examples of the type of data on AC and MDAC focusing on aspirin, acetaminophen, N-acetylcysteine, anticonvulsants, yellow oleander, isoniazid and theophylline as examples. This table gives the reader details on the extent and limitations of the clinical and animal data supporting the use of AC. A meta-analysis that included 64 controlled trials using AC in a variety of drug exposures in healthy volunteers found that AC was most effective when given within 1 h of the ingestion, but significant reductions in drug levels could still be seen even if given as long as 4 h after drug intake [98].

**Table 1:** Studies and reports evaluating the effects of activated charcoal on selected drugs

Type report	Subjects	Variables	Drug (oral unless noted)	Conclusion/reference
R, NB, CCS	HV	C, AC, VT	Acetaminophen	AC at 15, 30 and 120 min reduced acetaminophen urinary recovery by 48, 44, and 33% [127]

NR, OB, NB	HPP	AC + NAC, NAC	Acetaminophen	↓ In serum transaminases and prothrombin times with AC + NAC compared to NAC alone [128]

NR, OB, NB	HPP	AC + NAC, NAC	Acetaminophen	↓ In serum transaminase, major adverse effects and death with AC and NAC [129]

R, CCS, NB	HV	C, AC-after 1 h, AC-after 2 h, CL + AC-after 1 h	Acetaminophen	All significantly reduced acetaminophen AUC. The AUC was significantly more reduced when AC given at 1 h compared to 2 h, and GL did not add to AC alone [130]

R, NB	HPP	GL, AC, Ip, C	Acetaminophen	Greatest ↓ in acetaminophen level with AC, then Ip, then GL compared to C. No clinical differences reported [31]

NR, Retro, Ob, NB	HPP	GL + AC, AC, C	Acetaminophen	AC reduced risk for toxic acetaminophen concentrations, GL did not further reduce risk [131]

R, CCS, NB	HV	NAC, AC + NAC	NAC	No significant differences in peak NAC levels with AC [132]

NR, NB, CCS	HV	NAC, AC + NAC	NAC	A 3% reduction in NAC AUC and a 29% reduction in peak NAC levels with AC [133]

[	HV	AC, C	Acetaminophen	The acetaminophen AUC was 58.9% with AC compared to C (*P *= 0.01) [134]

Ob, NB	HPP	NAC, NAC + AC	Acetaminophen	The addition of AC significantly (*P *< 0.05) reduced the T 1/2 and increased the body clearance of acetaminophen [135]

B, R, CCS	HV	C, AC1, AC2 (variable types of AC)	Acetaminophen	Both types of AC reduced AUC for acetaminophen and peak levels of acetaminophen [136]

R, CCS, NB	HV	C, AC, AC+ IM atropine	Acetaminophen	AC significantly reduced AUC for acetaminophen by 20% alone and by 47% in the presence of atropine [137]

CCS, R, NB	Pigs	C, MDAC (variable times)	IV-acetaminophen, digoxin, theophylline, valproic acid	Significantly enhanced elimination (*P *< 0.01) for acetaminophen, theophylline and digoxin with MDAC, but no increased elimination with valproic acid [138]

R, CSS, NB	HV	C, AC (variable time after ingestion)	Acetaminophen + oxycodone	Compared to control, acetaminophen AUC reduced by 43% 1 h (*P *< 0.0001), 22% 2 h (*P *= 0.02) and 15% 3 h (*P *= 0.26) with AC [139]

R, CCS, NB	HV	C, AC (variable time after ingestion)	Acetaminophen	Compared to control, acetaminophen AUC reduced by 56% 1 h (*P *< 0.002), 22% 2 h (*P *< 0.03) and 8% 4 h (NS) with AC [140]

R, CSS, NB	HV	C, AC	Sodium amino-salicylic acid (1 and 2 g - C, 1 and 2 g - AC, 10 and 20 g - AC	AC was given immediately after salicylic acid. Increasing the dose of salicylic acid reduced the antidotal efficacy of AC and lead to increasing salicylic acid AUC. The salicylic acid AUC increased by more than 4 fold when salicylic acid 10 g dose went to 20 g dose with AC dose held constant [141]

R, CSS, NB	HV	AC (3 variable doses)	Acetaminophen	A 59% increase (*P *< 0.001) acetaminophen AUC was seen between 50 g AC and 5 g AC both given 1 h after drug [99]

R, CCS, NB	HV	C, Ip, GL, AC after 1 h	Aspirin	Equal reduction in absorption of aspirin as measured by recovered urine salicylate [142]

R, CCS, NB	HV	C, AC, MDAC (1, 2 or 3 doses separated by 4 h)	Aspirin	All 3 AC doses associated with significant (*P *≤ 0.01) reduction in urinary salicylate recovery. 3 doses of AC resulted in significantly (*P *< 0.01) greater recovery of salicylate than 1 or 2 AC doses [143]

R, CCS, NB	HV	C, AC	Aspirin	MDAC associated with a significant (*P *≤ 0.01) 9% reduction in serum salicylate AUC and a significant (*P *≤ 0.05) 18% reduction in urinary excretion. Considered "clinically modest" effect of "questionable valve" [144]

R, CCS, NB	HV	C, Ip, AC, Ip + AC	Aspirin	Urinary salicylate recovery was 96.3 ± 7.5% in control, 70.2 ± 12.1% Ip, 56.5 ± 12.5% AC, 72.7 ± 14.1%, Ip + AC. There was a significantly greater (*P *< 0.05) reduction with AC compared to Ip [18]

R, CCS, NB	HV	C, AC (1 h after ingestion)	Aspirin, digoxin, phenytoin	AC reduced the AUC of digoxin (98%), phenytoin (90%) and aspirin (70) [145]

CCS, NB	Canines	C, MDAC	IV-theophylline at 2 different doses	Nasogastric tube in duodenum, AC resulted in 22-47% decrease in theophylline AUC [146]

R, NB	Rats	C, AC, MDAC	IV-theophylline and phenobarbital	MDAC significantly decreased theophylline and phenobarbital serum T 1/2 and AUC while AC had only slight decrease. Thought to be "adsorption" of exsorbed theophylline and phenobarbital [147]

R, CCS, NB	HV	C, MDAC various doses, variable intervals for total dose 150 g AC	IV-theophylline	The AUC of theophylline significantly (*P *< 0.05) reduced near equally by three schedules of MDAC [148]

R, NB	Rats	C, MDAC	IV-theophylline multiple doses tested	The theophylline AUC and T 1/2 was reduced by 50% and 52% respectively by MDAC [149]

NB	HPP	MDAC	Phenytoin/phenobarbital	Apparent decreased T 1/2 for phenytoin and phenobarbital only after MDAC started [150]

NB	HPP	MDAC	Phenobarbital	Apparent decreased T 1/2 for phenobarbital with MDAC [151]

R, NB	HPP	MDAC, AC	Phenobarbital	In the 5 patients treated with MDAC, the T 1/2 was 36 ± 13 h for phenobarbital, significantly shorter than T 1/2 after single dose AC in 5 patients. No difference in length of time on mechanical ventilation or time in hospital [100]

NB	HPP	MDAC	Phenobarbital	Apparent decrease in T 1/2 phenobarbital with MDAC [152]

R, CSS, NB	HV	C, MDAC, 24 h of urinary alkalinization	IV-phenobarbital	The T 1/2 of phenobarbital was 148 h, 47 h and 19 h during the control, alkalinization and MDAC phases, respectively. All statistically significantly different from each other [153]

R, CSS, NB	HV	C, MDAC	IV-phenobarbital	MDAC deceased phenobarbital T 1/2 from 110 ± 8 to 45 ± 6 h (*P *< 0.01) [154]

NB	HPP	MDAC	Phenytoin	Apparent decrease in T 1/2 phenytoin with MDAC [155]

NB	HPP	MDAC	Phenytoin	Apparent decrease in T 1/2 phenytoin with MDAC [156]

R, CSS, NB	HV	C, MDAC	IV-phenytoin	MDAC decreased T 1/2 phenytoin from 44.5 to 72.3 h [157]

R, NB	HPP	AC, MDAC	Carbamazepine	MDAC associated with reduced T 1/2 carbamazepine 12.56 ± 3.5 vs. 27.88 ± 7.36 h (*P *= 0.0004) compared to single dose AC. MDAC also associated with statistically significant reduced coma, mechanical ventilation and length of hospital stay [101]

NB	HPP	MDAC	Carbamazepine	Apparent decrease in T 1/2 carbamazepine with MDAC [158]

NB	HPP	MDAC, WBI	Carbamazepine	Rebound in carbamazepine serum levels despite MDAC [45]

NB	HPP	MDAC	Valproic acid	Apparent decrease in T 1/2 valproic acid with MDAC [159]

R, NB	HPP	C, AC, MDAC	Pesticides, yellow oleander, medicines or unknown	No difference in rates of mortality between C (6.8%), AC (7.1%) and MDAC (6.3%). Odds ratio 0.96 (95% (F 0.70-1.33) between C and MDAC [104]

R, NB	HPP	C, MDAC	Yellow oleander	MDAC significantly (*P *= 0.025) reduced mortality from 8% (control) to 2.5% (MDAC). Significant reduction in ICU, digoxin FAB fragments treatment, cardiac pacing, life-threatening arrhythmias, doses of atropine and time in hospital with more [103]

R, CSS, NB	HV	C, AC	Isoniazid	AC reduced isoniazid absorption [160]

R, NB	Rabbits	C, AC	Isoniazid	AC reduced T 1/2 of isoniazid [161]

R, CSS, NB	HV	C, AC	Isoniazid	AC 1 h after isoniazid reduced the isoniazid AUC [162]

↑ = increased; ↓ = decreased; AC = activated charcoal; AUC = area under serum curve; B = blinded; C = control; CCS = crossover controlled study; GL = gastric lavage; HPP = human poisoned patients; HV = human volunteers; IM = intramuscular; Ip = ipecac; IV = intravenous; LiCl = lithium chloride; MC = multiple center; MDAC = multiple dose AC; NAC = n-acetylcysteine; NB = non-blinded; NC = no change; NR = non-randomized; ob = observational study; R = randomized; Retro = retrospective; SPS = sodium polystyrene sulfonate; SR = sustained release; T 1/2 = serum half-life; VT = variable time

Reduction in drug absorption also was significantly correlated with the AC/drug ratio (*P *= 0.02). Significant reductions in drug absorption at an AC to drug ratio of 10:1 were demonstrated, and little further reduction beyond the 100:1 ratio could be found. It was noted that drugs with a large volume of distributions demonstrated greater reductions in absorption with AC [98]. In vitro and in vivo studies have suggested that an AC to drug ratio of at least 10:1 for aspirin and acetaminophen should be obtained to insure maximal efficacy [99]. This recommendation on the AC to drug ratio is consistent with the findings of the above meta-analysis and has been generalized to all drugs with little additional clinical evidence [99].

Trials in actual poisoned patients with single dose AC or MDAC have shown mixed results. A randomized trial of ten patients who overdosed on phenobarbital reported a profound reduction in serum phenobarbital half-life, but no change in time on mechanical ventilation or the time in the hospital between those receiving a single dose of AC or MDAC [100]. In contrast, 12 patients with confirmed carbamazepine poisonings were randomized to receive MDAC or a single dose of AC [101]. Though peak carbamazepine levels were similar, the duration of coma was significantly decreased (*P *= 0.02) as were the hours on mechanical ventilation (*P *= 0.001) and the length of hospital stay (*P *= 0.00001) with MDAC. The serum half-life of carbamazepine was also significantly (*P *= 0.004) reduced by more than 50% with MDAC compared to a single dose of AC.

Merigian and Blaho [102] reported a prospective, randomized, controlled trial of AC compared to control (no treatment) after oral drug overdoses in patients who were able to take oral AC. Specific exposures were excluded, including ingestions of > 140 mg/kg acetaminophen, crack cocaine, mushrooms, volatiles, caustics, heavy metals, lithium and iron [102]. No GE was performed. Almost 1,500 patients were entered into the study and no difference in outcome parameters was seen between the two groups. In contrast, de Silva [103], randomized 401 patients who had ingested yellow oleander to MDAC or a single dose of AC. There were fewer deaths (2.5% vs. 8%, *P *= 0.025) for those treated with MDAC compared to a single dose of AC in this study. In a larger randomized trial that included 4,629 overdosed patients, Eddleston reported that there were no differences in mortality between control, single-dose AC and MDAC treatment in patients overdosed on various toxins (51% pesticides and 36% yellow oleander) [104]. A randomized trial of AC compared to control in 327 patients presenting with oral overdoses reported no significant reduction in hospital length stay or other patient outcomes with AC [105].

Compliance has been variable with AC even when ordered. In the previously mentioned large randomized controlled trials of control, AC and MDAC [104], a single dose of AC was only given 83% of the time, and only 66% of five doses of MDAC were actually given [106]. The major reasons for not receiving the AC doses were patient refusal and active vomiting. A descriptive study in pediatric poisonings found only 55% of children were given AC within 1 h of presentation to ED, and only 7.8% actually got their AC within 1 h of their poisoning because of delays in arriving to and delays within the ED [107]. Karim et al. [108] found that the median time to arrival after overdose was 136 min, and only 15 out of 63 patients received AC within 1 h of poisoning. A study in rural Australia reported the time from ingestion to ambulance arrival averaged 1 h 23 min, and the time to hospital averaged 2 h and 15 min [109]. They concluded that poisonings with long transport times would have to get AC in the ambulance if they were to have any chance of receiving AC within 1 h.

Many metals and electrolytes do not bind to AC, such as iron, lead, potassium and lithium [110]. The lack of significant binding of these agents to AC eliminates its potential usefulness in treating potassium, lithium, iron, lead and other heavy metal exposures.

Although AC is generally well tolerated, a number of complications have been reported with its use (Table 2). Many of these complications have been reported as case reports, and some are associated with MDAC, often as a result of the cathartic in the combined product. In a study of 575 poisoned patients treated with AC, adverse events occurred in 41 cases (7.1%) with nausea/vomiting found in 36, bronchoaspiration in 6 and pneumonia in 2 [111]. Spontaneous vomiting before AC, pre-hospital AC administration, repeated doses of AC and the need for specific clinical measures to treat intoxicated patients (e.g., intubation) were all associated with a significantly increased risk for an adverse event. A retrospective study in which 878 poisoned patients were treated with MDAC found that 5 (0.6%, 95% CI 0.1-1.1%) patients had clinically significant aspiration and none had GI obstruction [112]. No patients died. Mild hypernatremia (> 145 mEq/l) was seen in 53 patients (6.0%, 95% CI, 4.4-7.6%), with 5 of these patients having sodium levels greater than 155 meg/l. Hypermagnesemia (> 2.5 mg/dl) was seen in 27 patients (3.1%, 95% CI, 2.0-4.2%), and 3 patients had peak values greater than 3.75 mg/dl. These electrolyte abnormalities were usually associated with cathartic use, but not exclusively. One patient had a corneal abrasion that resolved without complication and was associated with AC getting into their eyes [112].

**Table 2:** Complications and Adverse Reactions to Activated Charcoal

Bronchiolitis obliteran after charcoal aspiration and Bronchopulmonary aspiration
Corneal abrasion
Fluid and electrolyte abnormalities¹
Hypernatremia
Hypermagnesemia
Gastrointestinal tract perforation/charcoal peritonitis

Nausea/vomiting
Pneumonia
Pneumothorax/charcoal-containing empyema
Small bowel obstruction with/without bezoar

1 = When AC given with a cathartic

In evaluating 50 intubated patients with evidence of new pulmonary infiltrates on chest X-ray within the first 48 h of hospitalization after AC treatment, only 2 (4%) had initial negative radiographs and then developed a new infiltrate after AC. This suggests an infrequent association of AC to aspiration pneumonia [113]. Severe cases of pulmonary aspiration of AC resulting in prolonged respiratory failure, death, empyema and bronchiolitis obliterans have been reported, but these are isolated case reports [114-117].

The risk factors for emesis after AC in poisoned patients were found to be prior vomiting before AC and the use of a nasogastric tube for AC administration. In a study of 275 children, 56 (20.4%) had vomiting after administration of AC [118]. Case reports of charcoal bezoars or inspissated charcoal being associated with small bowel obstruction exist after treatment with AC, but these events are also likely rare [119-122]. Acute appendicitis, charcoal stercolith associated with intestinal perforation and charcoal peritoneum has all been reported with AC treatment [123-126].

The last major practical issue in the decision to use charcoal for decontamination of a poisoned patient revolves around time of ingestion. The AACT/EAPCCT 1997 guidelines recommend that single dose AC should not be routinely administered to poisoned patients and suggest its effectiveness decreases with time after ingestion [7]. If charcoal is to be administered, the greatest benefit is seen within 1 h after ingestion of poison. There is no convincing clinical evidence that AC improves clinical outcome [7]. These 1997 recommendations were reaffirmed in 2005 with the observation that "no new evidence" was found to suggest a revision in the guidelines was needed [8]. When considering MDAC, the position paper from AACT/EAPCCT suggests that MDAC should only be considered in patients with protected or intact airways. MDAC should not be used with repeat doses of cathartics, and it should only be considered if a patient has ingested a life-threatening amount of carbamazepine, dapsone, phenobarbital, quinine or theophylline [9]. In 1995, 7.7% of all poisoned patients and 3.56% of all those patients ≤ 5 years old recorded by the AAPCC were treated with AC, but by 2009 the percentage had decreased to 3.4% of all poisoned patients and only 1.48% of patients ≤ 5 years old [27].

## Conclusion

Gastrointestinal decontamination with ipecac, GL, AC and cathartics are now used less often in the hospital setting in the poisoned patient. Whole bowel irrigation for the ingestion of slow-release medications and asymptomatic foreign body drug containers (body packers/stuffers) is recommended with little quality clinical data. Current recommendations for the use of AC and MDAC are limited in the treatment of the poisoned patient. The use of AC appears to be most efficacious when given within an hour of ingestion. The use of SPS as a binder of lithium is based on limited data. The current recommendations for GI decontamination of the poisoned patient are based on a few clinical trials, small case series, retrospective analysis and animal data. The previous aggressive approach to GI decontamination is increasingly being replaced by less emphasis on active GI decontamination and more emphasis on supportive care.

## Abbreviations

AACT: American Academy of Clinical Toxicology; AC: activated charcoal; AUC: area under the concentration curve; EAPCCT: European Association of Poison Centres and Clinical Toxicologists; ED: emergency department; GE: gastric emptying; GI: gastrointestinal; GL: gastric lavage; MDAC: multiple dose activated charcoal; NS: not statistically significant; PEG: polyethylene glycol; SPS: sodium polystyrene; WBI: whole bowel irrigation.

## Competing interests

The authors declare that they have no competing interests.

## Authors' contributions

All the authors participated in the literature search and evaluation of the literature. All authors participated in writing and editing of the manuscript.
